# The Risk Reduction Effect of a Nutritional Intervention With a Partially Hydrolyzed Whey-Based Formula on Cow's Milk Protein Allergy and Atopic Dermatitis in High-Risk Infants Within the First 6 Months of Life: The Allergy Reduction Trial (A.R.T.), a Multicenter Double-Blinded Randomized Controlled Study

**DOI:** 10.3389/fnut.2022.863599

**Published:** 2022-05-25

**Authors:** Nicolaos Nicolaou, Rouzha Pancheva, Eva Karaglani, Mikaela Sekkidou, Miglena Marinova-Achkar, Simoneta Popova, Margarita Tzaki, Anastasia Kapetanaki, Nicoletta Iacovidou, Theodora Boutsikou, Zoi Iliodromiti, Vassiliki Papaevangelou, Olympia Sardeli, Paraskevi Xepapadaki, Evangelia Papathoma, Inge Thijs-Verhoeven, Urszula Kudla, Laurien H. Ulfman, Anne Schaafsma, Yannis Manios

**Affiliations:** ^1^Asthma and Allergy Centre, Limassol, Cyprus; ^2^University of Nicosia Medical School, Nicosia, Cyprus; ^3^Department of Hygiene and Epidemiology, Faculty of Public Health, Medical University of Varna, Varna, Bulgaria; ^4^Department of Nutrition & Dietetics, School of Health Science & Education, Harokopio University, Athens, Greece; ^5^General Hospital Elena Venizelou, Athens, Greece; ^6^Neonatal Department, National and Kapodistrian University of Athens, Aretaieio Hospital, Athens, Greece; ^7^Third Department of Pediatrics, National and Kapodistrian University of Athens, ATTIKON General University Hospital, Athens, Greece; ^8^Allergy Department, 2nd Pediatric Clinic, National and Kapodistrian University of Athens, Athens, Greece; ^9^Neonatal Intensive Care Unit, Alexandra University and State Maternity Hospital, Athens, Greece; ^10^FrieslandCampina, Amersfoort, Netherlands; ^11^Institute of Agri-Food and Life Sciences, Hellenic Mediterranean University Research Centre, Heraklion, Greece

**Keywords:** nutritional intervention, high-risk infants, partially hydrolyzed formula, cow milk allergy, atopic dermatitis, allergy prevention, randomized controlled trial

## Abstract

**Background:**

The role of partially hydrolyzed formulas (pHF) as part of nutritional interventions to prevent the development of allergic manifestations (AM) is questioned, and efficacy of each specific pHF should be substantiated.

**Objective:**

To investigate the risk-reduction effect of a whey-based pHF on the development of cow's milk protein allergy (CMPA) and atopic dermatitis (AD) in infants at high-risk for allergy within the first 6 months of life.

**Materials and Methods:**

In a multicenter double-blinded randomized controlled setting, healthy non-exclusively breastfed full-term infants, received either a specific whey-based pHF or a standard cow's milk-based formula (SF) and were clinically assessed for AM at 2, 4, and 6 months of age, supported by the objective scoring tools SCORAD and CoMiSS. CMPA was confirmed by open food challenge. Intention-to-Treat (ITT) and Per-Protocol (PP) analyses were performed.

**Results:**

Of 331 randomized subjects (ITT analysis set), 160 received the pHF and 171 the SF. Six (3.8%) infants in the pHF and 12 (7%) in the SF group developed CMPA (*p* = 0.186). AD incidence was significantly lower in those receiving pHF as compared to SF (10.6% vs. 18.7%, *p* = 0.024) with a relative risk (RR, 95% CI) of 0.54 (0.32, 0.92), in particular when adjusting for family history of AD [6.5% vs. 27.3%, RR 0.24 (0.07, 0.78), *p* = 0.018] representing a risk reduction of 76%. The PP analysis showed similar results.

**Conclusion:**

This specific whey-based pHF reduced the risk of AD development, particularly in those with a family history of AD, and tended to reduce the development of CMPA in non-exclusively breastfed infants at high-risk for allergy. The A.R.T. study suggests that this particular pHF may contribute to measures aimed at prevention of allergic manifestations. However, further studies are needed to confirm this risk-reduction effect.

## Introduction

Over the last few decades Cow's Milk Protein Allergy (CMPA) and Atopic Dermatitis (AD) have become significant worldwide health problems in early life, affecting approximately 0.5–3% and 15–20% of the pediatric population, respectively (1, 2). Both are commonly considered as leading steps in the “Atopic March” followed by further allergic manifestations (AM), in particular other food allergies, asthma and rhinitis. In an effort to halt this progress in allergic phenotypes, several prevention strategies including nutritional interventions have been proposed (3–5).

Within this concept, partially hydrolyzed formulas (pHF) have been suggested as means to prevent the development of allergic manifestations in high-risk infants who are not exclusively breastfed (6, 7). A number of studies including meta-analyses and systematic reviews were indicative of a risk-reducing effect of pHF particularly for AD (8–12). However, the role of pHF has recently been questioned based on the lack of robust scientific evidence supporting their efficacy in allergy prevention (13, 14). Beyond differences in study designs, hydrolysates used and methodological limitations, concerns for conflict of interest and publication bias have also been raised, as research in this field is usually sponsored by milk formula manufacturers (7, 14).

It is important to note that not all hydrolyzed formulas are the same in terms of allergenicity and tolerance induction capacity (depending on the milk protein processing and degree of hydrolysis) which may reflect the differences observed in their effectiveness in allergy prevention (15–20). In 2016 the European Food Safety Authority (EFSA) stated (21) that the safety, suitability and efficacy of each specific formula containing protein hydrolysate(s) has to be established by clinical trials implementing high methodological rigor. Addressing the recommendation by EFSA, the main aim of the A.R.T. study was to investigate the potential role of a nutritional intervention with a partially hydrolyzed whey-based formula (Frisolac Gold preventive HA) compared to a standard formula (Frisolac Gold) in reducing the incidence of CMPA and AD in non-exclusively breastfed infants at high-risk for allergy within the first 6 months of life.

## Methods

### Study Design and Participants

The Allergy Reduction Trial (A.R.T.) is a multicenter, double-blinded, parallel, randomized controlled study assessing differences in the incidence of CMPA [presented as part of milk related allergic manifestations (MRAM) in the original study protocol] and AD within the first 6 months of life in apparently healthy term infants at high-risk of developing allergy (family history of allergy), conducted in 6 centers in 3 countries; Bulgaria (1), Cyprus (1) and Greece (4) between 2017–2019. Two intervention formulas were provided, either to be fed exclusively or supplementary to breastfeeding: a) a partially hydrolyzed whey-based formula (pHF) or b) an intact protein (standard) cow's milk formula (SF) containing both whey and casein fractions in a 60:40 ratio. Exclusively breastfed infants were followed as a parallel observational group.

Only term infants (≥37 weeks), with birth weight ≥2500g, postnatal age <5 days, apparently healthy with no signs of allergy, who were exclusively breastfed or fed with an extensively hydrolyzed infant formula (eHF) since birth were recruited. The inclusion criteria are detailed in the Supplementary Material (SM).

The study protocol, information letter to parents/legal guardians, and written informed consent form were approved by the appropriate independent ethics committee in each center as detailed in the SM. The study was conducted in accordance with the guidelines of the Declaration of Helsinki and the International Conference on Harmonization guidelines on Good Clinical Practice and registered in the Netherlands Trial Registry [Identifier: Trial NL6120 (NTR6259)].

### Recruitment, Randomization and Treatment Allocation

During the 7^th^-9^th^ month of gestation (or shortly after delivery), families attending public and private maternity clinics, were interviewed regarding family history of allergy (Atopic Dermatitis/Eczema, Allergic Asthma, Allergic Rhinitis-Conjunctivitis, Urticaria and Food Allergy), using an enriched validated questionnaire (10) to identify infants at high-risk of developing allergy (at least one parent or sibling with doctor-diagnosed allergy). Within 4 days after delivery, parents willing to participate in the study signed the informed consent form.

All mothers were strongly encouraged to breastfeed and inclusion in the study was not dependent on mothers' choice of feeding regimen. In case of non-exclusive breastfeeding, infants were randomly allocated to the pHF or the SF based on computer-generated randomization tables with stratification for gender, type of feeding [exclusively formula-fed or mixed-fed (breastfeeding combined with infant formula)] and presence of AD in the family. Upon parents' request, infants from the exclusively breastfed group could be allocated to one of the two formula groups within 10 weeks of life.

### Study Products, Blinding and Adverse Events

The intervention formulas were a partially hydrolyzed whey-based formula (pHF; Frisolac Gold preventive HA) and an intact protein/standard formula (SF; Frisolac Gold) nutritionally suitable for the first 6 months of life (Supplementary Table S1). Both formulas were produced in the Netherlands by FrieslandCampina and packed in identical white unlabeled 400g tins with the code-name of each formula group (FCA and FCB) at the bottom. This was performed by the packaging department at FrieslandCampina not involved in the study. All study personnel, FrieslandCampina employees involved in the study and parents/legal guardians were blinded to the study formulas until the whole study was completed and database was locked.

All adverse events and actions taken were recorded throughout the study and monitored by an independent Data Safety Monitoring Board (DSMB).

### Follow-Up Evaluation and Compliance

After allocation, infants visited the study centers for bi-monthly follow-up assessments (at 2^nd^, 4^th^, and 6^th^ month) during the first 6 months of life. Additional visits were performed if needed, such as in case of development of any signs of allergy or adverse events. Infants had not consumed any other formula with intact or partially hydrolyzed protein prior to allocation and solid foods were allowed after the age of 4 months. No dietary restrictions were advised to breastfeeding mothers. Formula intake compliance was evaluated using a 7-day milk diary completed the week preceding the 1^st^, 2^nd^, 4^th^, and 6^th^ month of age. If formula consumption was <40 mL/kg body weight/day during the 1^st^ month or <60 mL/kg body weight/day during the 2^nd^ month (and thereafter), the infant was considered as a drop-out (22).

At follow-up visits, infants were clinically examined by experienced pediatricians and nurses for the presence of CMPA and AD. Suggestive CMPA and AD symptoms were objectively scored using the Scoring for Atopic Dermatitis [SCORAD (23)] tool and the awareness Cow's Milk-related Symptom Score [CoMiSS (24, 25)] tool. Furthermore, the “Screening for IgE- and non-IgE-mediated food allergy symptoms questionnaire” was completed (26).

Anthropometric measurements (weight, length, and head circumference) were performed by the same two well-trained research team members at each center, using calibrated digital infant scales (SECA 354), infantometers (SECA 210) and non-elastic tapes (SECA 211) respectively. All measurements were performed in triplicates and averaged.

### Definition of Study Outcomes

#### Primary Outcomes

Two primary outcomes were defined: CMPA (as part of MRAM) and AD.

#### Cow's Milk Protein Allergy

CMPA was confirmed by CMP elimination diet (in selected cases accompanied by skin prick tests) followed by a positive oral food challenge (OFC) at any time-point during the first 6 months of life.

*Specifically, when an infant presented with any symptoms/signs suggestive of either IgE- or non-IgE-mediated CMPA (e.g. urticaria, angioedema, AD, vomiting, diarrhea, blood in stools), the EAACI* (6) *and USA* (27) *guidelines were followed to confirm the diagnosis. The first step was always a thorough medical history and physical examination in combination with recorded SCORAD, CoMiSS tools and the IgE- and non-IgE symptoms questionnaire. If symptoms could not be explained by another cause, CMPA was considered as a potential diagnosis and a CMP (cow's milk protein) elimination diet was initiated for 7–14 days. Disappearance of symptoms was followed by an OFC*.

*In case of mild non-IgE symptoms, the pediatrician performed the open OFC. Symptoms were first treated with an eHF or an AAF (when symptoms did not disappear), for at least seven days. After disappearance of the symptoms, reintroduction of the study formula would take place at home or in an outpatient setting. In case of a positive OFC outcome (reappearance of symptoms) the infant was diagnosed with CMPA. In case the OFC was negative, the infant could continue with the study formula. If the outcome of the challenge was inconclusive a DBPCFC had to be performed by the pediatric allergist*.

*In case of moderate/severe non-IgE or IgE-mediated suspected CMP-related allergic reactions, the infant was referred to the pediatric allergist for further assessment (skin prick tests and OFC with the study formula) in a controlled hospital setting. Prior to further testing the infants should have been in good general condition, the use of any antihistamines or glucocorticosteroids were discontinued and any AD was stabilized in the preceding week. In case of an inconclusive OFC outcome, a DBPCFC had to be performed*.

#### Atopic Dermatitis

AD was defined as the clinical diagnosis by the pediatrician (typical morphology and distribution of skin lesions, head, neck, trunk and extensor surface of the extremities) and extend and severity were objectified with the recorded SCORAD (total objective score ≥ 1) in combination with the supportive awareness tool CoMiSS (score for Skin Symptoms on Atopic Eczema ≥ 1).

#### Amendment of the Definition of the Primary Outcomes

*The A.R.T. study design and protocol were based on previously conducted studies in this research field* (10, 28) *which used as main outcome allergic manifestations (AM; including CMPA, AD, asthma, rhinitis and other food allergies). This definition of AM and MRAM were also used in the A.R.T. original study protocol. The previous studies reported outcomes at an older age of 6 months where other allergic manifestations i.e., asthma, rhinitis and other food allergies may also be present. However, within the first 6 months of life (time point at which A.R.T. reported outcomes) CMPA (as part of MRAM) and AD are the most common allergic manifestations developed. This is the main reason why we amended the original protocol outcome definitions and used only CMPA (confirmed by OFC) and AD as our primary outcomes*.

*Based on the study protocol and statistical analysis plan, the results of the present study were initially analyzed using the following definition for AD: “clinical diagnosis by the pediatrician (typical morphology and distribution of skin lesions, head, neck, trunk and extensor surface of the extremities) in combination with the recorded SCORAD (total score ≥ 1)”. Those results revealed a relatively high incidence for AD more likely because of overlapping skin manifestations in this early age (26.3% in the pHF group and 31.0% in the SF group) and parental reporting on sleep loss in section C on SCORAD without skin rash which was clinically irrelevant. Therefore, the A.R.T. study team decided to revise the definition used for AD including information on atopic eczema from the awareness CoMiSS tool in order to decrease the possibility of misreporting for AD. Of note, statistical analysis results on AD with initial AD definition also showed a significant risk reduction effect for the pHF mixed-fed group compared to the SF mixed-fed group*.

*The relevant official memo signed by all A.R.T. PIs and the sponsor is available upon request*.

#### Secondary Outcomes

Secondary outcomes included growth parameters (weight, length, head circumference) within the first 6 months of life, assessed and compared with World Health Organization growth charts (29). These data will be presented in detail in another manuscript.

### Sample Size Estimation and Statistical Analysis

The sample size calculation was based on two studies: the German Infant Nutritional Intervention (GINI) study (2003) (10), and the study performed by Halken et al. (2020) (28, 30). Assuming a drop-out rate of 30%, 158 infants had to be included per treatment arm. The detailed sample size estimation is presented in the SM.

Statistical analysis was carried out by an independent statistical company (OCS Life Sciences, The Netherlands) using the SAS software version 9.4 (SAS Institute, Cary, NC). Correction for multiple testing was done for the primary parameters by correcting the threshold using the Hochberg procedure (31).

Next to primary outcomes, *post-hoc* analysis was performed for CMPA defined as: (1) CMPA+_SPT_ including also IgE-mediated CMPA cases considered as CMPA based on clinical history and positive Skin Prick Test (SPT) to cow's milk without performing OFC and (2) CMPA+_susp_ including confirmed CMPA and CMPA+_SPT_ cases plus the clinically suspected cases of CMPA in which symptoms resolved after introducing an extensively hydrolyzed formula, but parents did not consent for OFC.

CMPA (confirmed by OFC) and AD outcomes were analyzed in the Intention-to-Treat (ITT) and Per-Protocol (PP) population. The ITT analysis set includes all study participants who were randomized to one of the study formulas (pHF or SF). The PP analysis set includes only the subjects in which no protocol deviation was recorded until the age of 6 months. Missing data were not imputed. For the ITT analysis the last available follow-up data were taken into account.

The incidence of CMPA and AD within the first 6 months of life were calculated and Relative Risks, 95% Confidence Interval (RR, 95% CI) were estimated separately by a Poisson General Equation Estimation (GEE) regression analysis (with a log link) applying two models. The first model adjusted for study formula and stratification factors (center, gender and family history of AD). The second model additionally adjusted for the interaction between family history of AD and study formula. A two-sided statistical significance level of *p* < 0.05 was accepted. The Number Needed to Treat (NNT) for AD as described by Sackett and Haynes (32) was also calculated.

## Results

### Study Population

**Figure 1:**
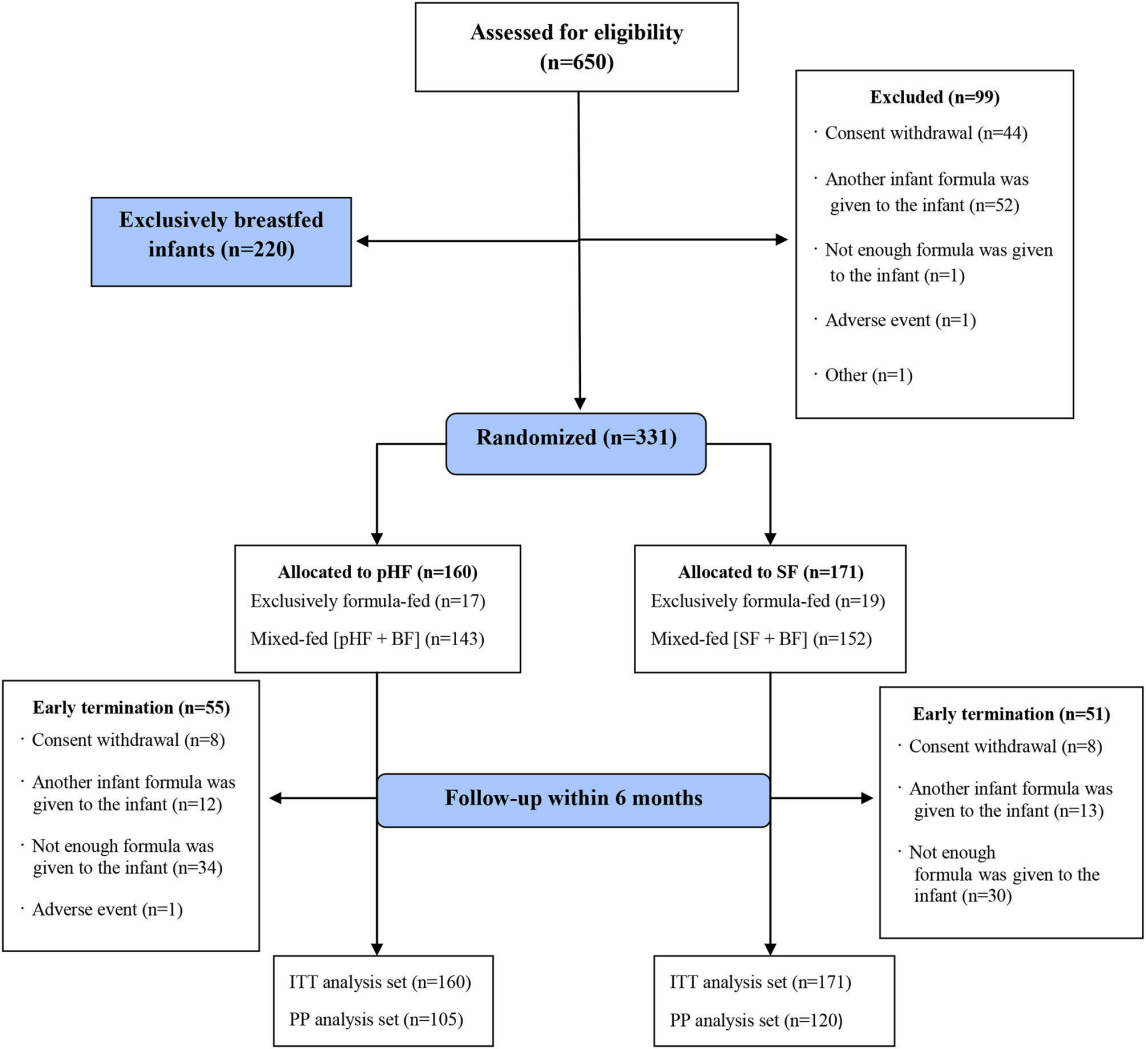
Flow diagram of A.R.T. study population. SF, standard formula; pHF, partially hydrolyzed formula; BF, breastfeeding; ITT, intention-to-treat analysis set; PP, per-protocol analysis set.

Of 650 subjects eligible for participating in the study, 99 were excluded before being assigned to any group, 331 were randomized to one of the two study formula groups and 220 were exclusively breastfed and followed as an observational group. The data for the exclusively breastfed infants will be presented in detail in another publication. The whole study population and reasons for early termination (dropouts) are presented in Figure 1. Of those randomized (ITT), 160 (48%) infants were allocated to the pHF (17 exclusively formula-fed, 143 mixed-fed) and 171 (52%) to the SF group (19 exclusively formula-fed, 154 mixed-fed). The PP analysis set consisted of 105 (66%) infants in the pHF group and 120 (70%) in SF group that strictly adhered to the study protocol.

The effect of pHF on the incidence and relative risk for CMPA and AD compared to SF is presented in 3 population groups: a) all formula-fed (exclusively and mixed-fed) b) mixed-fed and c) exclusively formula-fed infants.

### Baseline Characteristics of Study Participants

The baseline characteristics (including mode of delivery, birth weight, gender and pet ownership) of study participants for the pHF and SF groups are presented in Table 1. There were no significant differences in any of the characteristics between the two groups in both the ITT and PP sets, except from the residence in urban areas (ITT). In the ITT analysis set, for each participating country, the percentage of infants receiving the intervention and control formulas was almost identical (Bulgaria; 47.5 vs. 46.8%, Cyprus; 34.4 vs. 35.7%, Greece; 18.1 vs. 16.4% *p* = 0.73). Approximately 1 in 4 infants in each formula group had a family history of AD (pHF: 28.8% vs. SF: 25.7% *p* = 0.54).

**Table 1:** Baseline characteristics of study participants as in the ITT and PP analysis sets.

	**ITT analysis set**				**PP analysis set**			
	**pHF**	**SF**	***p*** -value^*^	**Total**	**pHF**	**SF**	***p*** -value^*^	**Total**
	**(*N =* 160)**	**(*N =* 171)**		**(*N =* 331)**	**(*N =* 105)**	**(*N =* 120)**		**(*N =* 225)**
**Country of study center, *n* (%)**								
***Bulgaria***	76 (47.5)	82 (48.0)	0.935	158 (47.7)	45 (42.9)	58 (48.3)	0.579	103 (45.8)
***Cyprus***	55 (34.4)	61 (35.7)		116 (35.0)	45 (42.9)	43 (35.8)		88 (39.1)
***Greece***	29 (18.1)	28 (16.4)		57 (17.2)	15 (14.3)	19 (15.8)		34 (15.1)
Infant characteristics								
**Normal conception, *n* (%)**	148 (93.1)	164 (95.9)	0.139	312 (94.5)	97 (93.3)	113 (94.2)	0.498	210 (93.8)
**Gestational age, weeks, mean (SD)**	38.7 (1.2)	38.7 (1.0)	0.744	38.7 (1.1)	38.6 (1.2)	38.7 (1.0)	0.389	38.7 (1.1)
**Cesarean delivery, *n* (%)**	106 (66.3)	106 (62.0)	0.42	212 (64.0)	69 (65.7)	73 (60.8)	0.45	142 (63.1)
**Birth weight, g, mean (SD)**	3,270.5 (433.6)	3,278.1 (453.7)	0.88	3,274.5 (443.4)	3,257.1 (431.4)	3,246.7 (449.6)	0.86	3,251.6 (440.2)
**Gender, female, *n* (%)**	67 (41.9)	78 (45.6)	0.493	145 (43.8)	47 (44.8)	55 (45.8)	0.87	102 (45.3)
**Family history of allergy**								
***Parents, n (%)***	139 (86.9)	143 (83.6)	0.406	282 (85.2)	88 (83.8)	102 (85.0)	0.806	190 (84.4)
***Siblings, n (%)***	40 (25.0)	48 (28.1)	0.654	88 (26.6)	32 (30.5)	33 (27.5)	0.374	65 (28.9)
**Family history of atopic dermatitis, n (%)**	46 (28.8)	44 (25.7)	0.537	90 (27.2)	26 (24.8)	34 (28.3)	0.546	60 (26.7)
Maternal characteristics								
**Maternal age, years, mean (SD)**	31.7 (5.1)	31.3 (5.1)	0.47	31.5 (5.1)	31.3 (4.8)	31.5 (5.4)	0.84	31.4 (5.1)
**Maternal education, *n* (%)**								
***≤14 Years***	60 (37.5)	70 (40.9)	0.522	130 (39.3)	43 (41.0)	52 (43.3)	0.718	95 (42.2)
***>14 Years***	100 (62.5)	101 (59.1)		201 (60.7)	62 (59.0)	68 (56.7)		130 (57.8)
**Maternal smoking in pregnancy, *n* (%)**	15 (9.4)	22 (12.9)	0.31	37 (11.2)	10 (9.5)	18 (15.0)	0.21	28 (12.4)
Paternal characteristics								
**Paternal age, years, mean (SD)**	34.5 (5.1)	34.1 (5.1)	0.558	34.3 (5.1)	34.4 (4.8)	34.1 (5.4)	0.758	34.2 (5.1)
**Paternal education, n (%)**								
***≤14 Years***	76 (47.8)	95 (55.6)	0.159	171 (51.8)	53 (51.0)	67 (55.8)	0.466	120 (53.6)
***>14 Years***	83 (52.2)	76 (44.4)		159 (48.2)	51 (49.0)	53 (44.2)		104 (46.4)
**Paternal smoking, *n* (%)**	77 (48.1)	83 (48.5)	0.94	160 (48.3)	55 (52.4)	54 (45.0)	0.269	109 (48.4)
**Urban residence, *n* (%)**	146 (91.3)	141 (82.9)	**0.025**	287 (87.0)	96 (91.4)	102 (85.7)	0.183	198 (88.4)
**Presence of pets, *n* (%)**	44 (27.7)	64 (37.4)	0.059	108 (32.7)	35 (33.7)	43 (35.8)	0.733	78 (34.8)
***Pet indoor, n (%)***	31 (70.5)	38 (60.3)	0.21	69 (64.5)	24 (68.6)	28 (66.7)	0.62	52 (67.5)

* *p-values for categorical variables are derived from Chi-Square or Fishers Exact Test*.

*p-values for birthweight and age of parents were calculated using Independent Samples T-test and for gestational age by using Wilcoxon Rank Sum Test. Figures in bold indicate statistically significant p-values. pHF, partially hydrolysed formula; SF, standard formula; PP, per-protocol; ITT, intention-to-treat; N, number of subjects in analysis population; n, number of non-missing observations; SD, Standard Deviation*.

### Amount of Infant Formula Consumed

The mean daily amount of infant formula (mL) consumed was similar between the two study formula groups at all follow-up visits (2^nd^, 4^th^, and 6^th^ month of age) in both the Per-Protocol and Intention-to-Treat analyses (Supplementary Table S4).

### Oral Food Challenges

Sixteen infants in the pHF group had symptoms/signs suggestive of CMPA and were offered an OFC. Six challenges were positive and 10 negative. In the SF group, 20 infants had suggestive CMPA symptoms/signs and were offered an OFC of which 12 challenges were positive, 5 negative and in 3 were never performed because of parental refusal.

### Effect of the pHF on CMPA and AD in All the Formula-Fed Infants

The incidence and RR for CMPA and AD within the first 6 months of life in formula-fed infants (both exclusively formula-fed and mixed-fed) in both analyses are presented in Table 2.

**Table 2:** The incidence and relative risk for CMPA and AD within the first six months of life in both exclusively formula-fed and mixed-fed infants.

			**ITT analysis set**				**PP analysis set**			
			**pHF**	**SF**	**RR (95% CI)**	***p*** **-value**	**pHF**	**SF**	**RR (95% CI)**	***p*** **-value**
			**(*N =* 160)**	**(*N =* 171)**			**(*N =* 105)**	**(*N =* 120)**		
**Model 1**		**CMPA, *n* (%)**	6 (3.8)	12 (7.0)	0.53 (0.21, 1.36)	0.19	5 (4.8)	10 (8.3)	0.63 (0.24, 1.70)	0.36
		**AD, *n* (%)**	17 (10.6)	32 (18.7)	0.54 (0.32, 0.92)	**0.024**	12 (11.4)	29 (24.2)	0.49 (0.26, 0.90)	**0.021**
**Model 2**	**FHAD +**	**n**	**46**	**44**			**26**	**34**		
		**CMPA, *n* (%)**	2 (4.3)	6 (13.6)	0.35 (0.08, 1.62)	0.18	2 (7.7)	4 (11.8)	0.74 (0.16, 3.38)	0.69
		**AD, *n* (%)**	3 (6.5)	12 (27.3)	0.24 (0.07, 0.78)	**0.018**	2 (7.7)	10 (29.4)	0.28 (0.07, 1.19)	0.085
	**FHAD -**	**n**	**114**	**127**			**79**	**86**		
		**CMPA, *n* (%)**	4 (3.5)	6 (4.7)	0.71 (0.21, 2.39)	0.58	3 (3.8)	6 (7.0)	0.58 (0.15, 2.16)	0.41
		**AD, *n* (%)**	14 (12.3)	20 (15.7)	0.74 (0.40, 1.37)	0.34	10 (12.7)	19 (22.1)	0.58 (0.29, 1.15)	0.12

*Poisson generalized estimating equation (GEE) regression analysis. Model 1 adjusts for study formula and stratification factors: country, gender and FHAD. Model 2 additionally adjusts for the interaction between FHAD and study formula. Figures in bold indicate statistically significant p-values. AD, atopic dermatitis; CMPA, cow's milk protein allergy confirmed by oral food challenge (OFC); pHF, partially hydrolysed formula; SF, standard formula; ITT, intention-to-treat; PP, per-protocol; RR, relative risk; FHAD+, Family history of AD; FHAD-, No family history of AD*.

In ITT analysis, 6 (3.8%) subjects in the pHF vs. 12 (7.0%) in the SF group developed CMPA within the first 6 months of life (*p* = 0.19). Adjusting for the interaction between FHAD and study formula did not provide any statistical significance. Similar results were observed for both Models in the PP analysis.

The incidence of AD was significantly lower in the pHF compared to SF group in both analyses (ITT: 10.6 vs. 18.7% *p* = 0.024, PP: 11.4 vs. 24.2% *p* = 0.021), with RR 0.54 (0.32, 0.92) and 0.49 (0.26, 0.90) respectively. The effect was stronger when adjusting for family history of AD (Model 2) as shown in ITT analysis [6.5 vs. 27.3%, RR 0.24 (0.07, 0.78), *p* = 0.018] representing a risk reduction effect of 76%. A trend was observed in PP analysis [7.7 vs. 29.4%, RR 0.28 (0.07, 1.19), *p* = 0.085].

### Effect of the pHF on CMPA and AD in Mixed-Fed Infants

**Figure 2:**
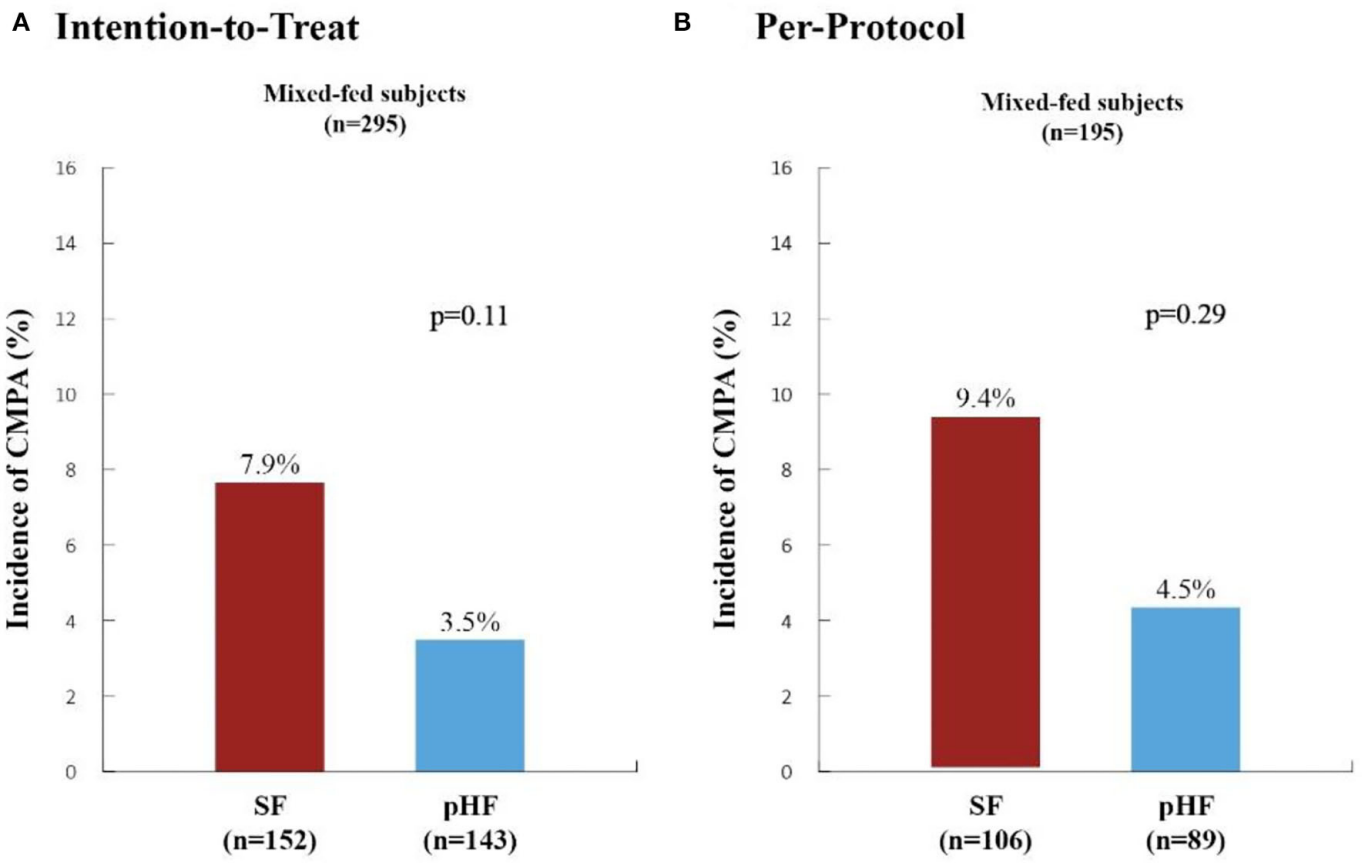
Incidence of Cow's Milk Protein Allergy within the first six months of life in high-risk mixed-fed infants. The incidence of cow's milk protein allergy (CMPA) in high-risk mixed-fed infants within the first 6 months of life as generated from Poisson generalized estimating equation (GEE) regression analysis (adjusting for study center, gender and type of formula) are presented in the Intention-to-Treat **(A)** and Per-Protocol **(B)** Analyses. SF, standard formula (red columns); pHF, partially hydrolyzed formula (blue columns). The incidence of CMPA observed in the SF group compared to the pHF was higher in both analyses (ITT: 7.9 vs. 3.5%, *p* = 0.11 and PP: 9.4 vs. 4.5%, *p* = 0.29) although the difference did not reach statistical significance.

The incidence of CMPA within the first 6 months of life in mixed-fed subjects for both analyses is depicted in Figure 2. The incidence of CMPA in the SF compared to the pHF group was higher in both analyses (ITT: 7.9 vs. 3.5%, *p* = 0.11 and PP: 9.4 vs. 4.5%, *p* = 0.29) although the difference did not reach statistical significance. *Post-hoc* analysis using CMPA+_SPT_ and CMPA+_susp_ showed a trend in the ITT analysis toward a preventive effect of pHF as compared to SF [CMPA+_SPT_: 4.2 vs. 9.2%, RR 0.45 (0.18, 1.12), *p* = 0.084, CMPA+_susp_: 4.9 vs. 10.5%, RR 0.46 (0.20, 1.11), *p* = 0.067].

**Figure 3:**
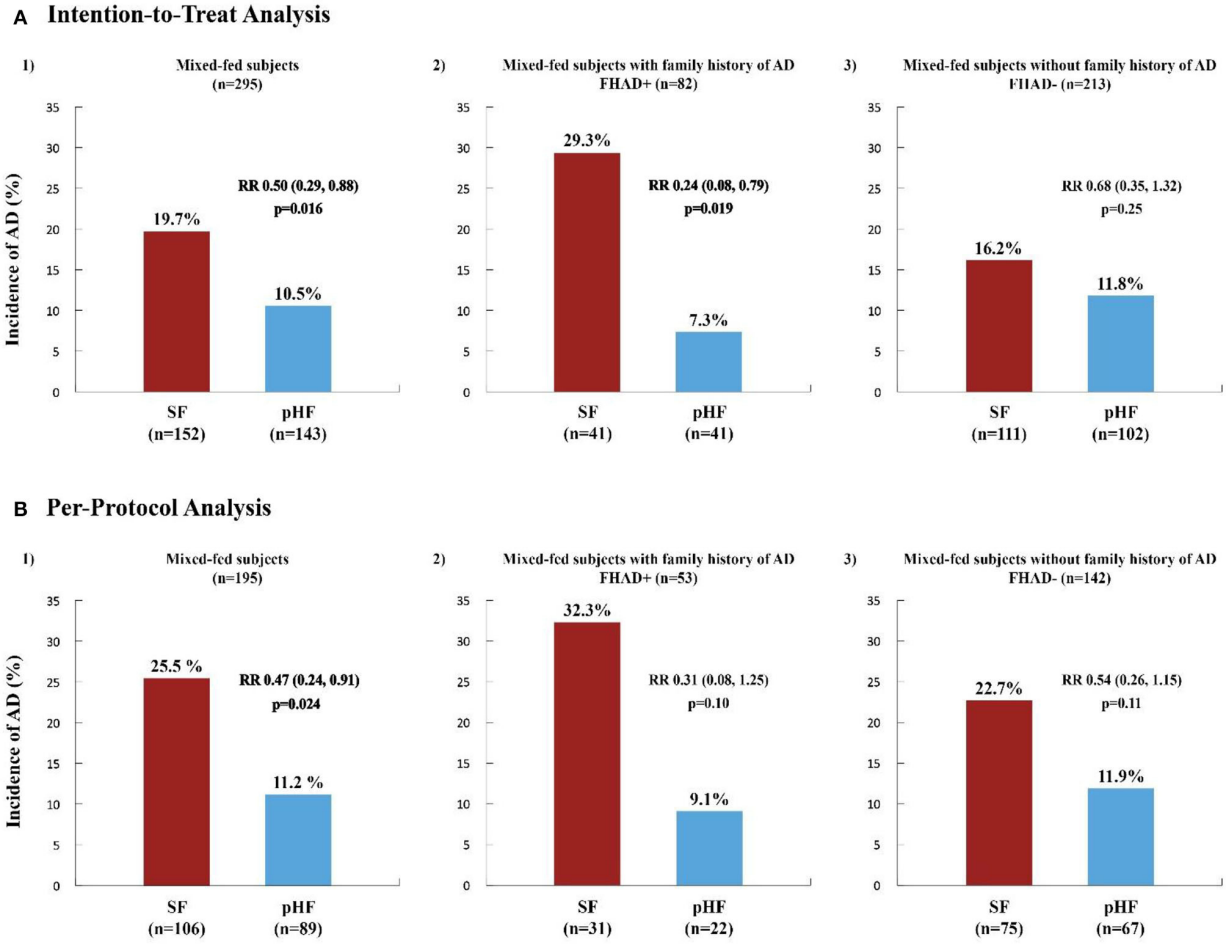
Incidence and relative risk of atopic dermatitis within the first 6 months of life in high-risk mixed-fed infants. The incidence and relative risk (RR) of Atopic Dermatitis (AD) in high-risk infants within the first 6 months of life, in the whole mixed-fed population and in subjects mixed-fed with (FHAD+) and without (FHAD-) family history of AD, generated from Poisson generalized estimating equation (GEE) regression analysis are presented in the Intention-to-Treat (ITT) **(A)** and Per-Protocol (PP) **(B)** Analysis. SF, standard formula (red columns); pHF, partially hydrolyzed formula (blue columns). A significant relative risk (RR, 95% CI) and risk reduction effect (%) of the pHF on the incidence of AD for the whole mixed-fed population was observed in both the ITT **(A1)** [RR 0.50 (0.29, 0.88), *p* = 0.016] and PP **(B1)** analysis [RR 0.47 (0.24, 0.91), *p* = 0.024], representing a risk reduction of 50% and 53% respectively. The reduction effect was most significant in those mixed-fed subjects with FHAD+ [RR 0.24 (0.08, 0.79), *p* = 0.019] receiving the pHF, representing a 76% reduction on the incidence of AD **(A2)**. In the PP analysis **(B2)** only a trend was shown in this study group (*p* = 0.10) whereas, no effect was observed for those infants without a family history of AD **(A3, B3)**.

Figure 3 illustrates the incidence and RR for AD in the same group. The incidence of AD was significantly lower in the pHF compared to the SF group in both analyses (ITT: 10.5 vs. 19.7% *p* = 0.016, PP: 11.2 vs. 25.5% *p* = 0.024), with RR 0.50 (0.29, 0.88) and 0.47 (0.24, 0.91), respectively. The effect was stronger when adjusting for family history of AD (Model 2) as shown in the ITT analysis [7.3 vs. 29.3%, RR 0.24 (0.08, 0.79), *p* = 0.019] representing a risk reduction effect of 76%. The calculated number needed to treat (NNT) (32) was 4.5, which means that the number of infants needed to be given the pHF in order to prevent the development of AD in 1 subject was 5. In the PP analysis, in Model 2 only a trend for AD was observed [9.1 vs. 32.3%, RR 0.31 (0.08, 1.25), *p* = 0.10].

The results for this group of infants are presented in detail in Supplementary Table S2.

### Effect of pHF on CMPA and AD in Exclusively Formula-Fed Infants

Given the small number of subjects exclusively formula-fed (pHF *n* = 17, SF *n* = 19) no differences could be detected (Supplementary Table S3).

## Discussion

### Principal Findings

In this multicenter double-blinded randomized controlled trial of non-exclusively breastfed high-risk for allergy infants, nutritional intervention with a partially hydrolyzed whey-based formula compared to a standard formula resulted in a significant reduction in the cumulative incidence of atopic dermatitis within the first 6 months of life. The risk reduction effect was strongest (76%) in mixed-fed infants with a family history of AD. In this group, the number of subjects needed to receive the specific pHF in order to prevent the development of 1 case of AD was 5. Although the incidence of CMPA as confirmed by OFC in the SF compared to the pHF group was two times higher (12 vs. 6 cases; 7 vs. 3.8% in the whole study population), the difference did not reach statistical significance. However, clinical significance and a potential preventive role of pHF in CMPA may not be excluded.

### Infant Feeding Practices

Breastfeeding is undoubtfully the ideal way of feeding in early life and should be strongly encouraged (33–35). However, in real life, not all infants are exclusively breastfed and a number of recent studies have shown that mixed-feeding is a commonly practiced form of nutrition (36–38). In the present study, approximately 20% of the mothers who initially expressed their willingness to exclusively breastfeed, introduced another formula as supplementary feeding at some point during the study. The fact that the majority of infants (90%) in A.R.T. who were randomized to any of the 2 study formulas had also received breastmilk during the first 6 months of life, further supports this notion and reflects the successful efforts of breastfeeding campaigns (39–41). Mixed feeding may have an additional beneficial effect in the prevention of CMPA as shown in a large prospective USA study which demonstrated that the combination of breastmilk and infant formula feeding is associated with the lowest rate of food protein-induced allergic proctocolitis, the most common CMPA clinical presentation in the first year of life (42).

### The Debatable Role of pHF in Allergy Prevention

Partially hydrolyzed formulas have been used as a nutritional intervention to prevent the development of allergic manifestations in non-exclusively breastfed high-risk infants (7, 43–45). This recommendation was mainly based on the findings of studies reporting a reducing effect particularly on the incidence of AD (9, 10, 28, 46). The GINI study (10, 47–50), considered as the reference study in this research area, showed a preventive role of a specific pHF on AD, up to the age of 15 years, but not at all follow-up time points. In GINI, the significant reduction effect in AD was observed in infants without a family history of AD, whereas in A.R.T. the reduction effect was strongest in those infants with a family history of AD. This may be explained by methodological differences between the two studies including that in A.R.T. formula introduction was allowed until the age of 10 weeks and AD outcome reported at 6 months of age, whereas in GINI the formula was introduced at any time point during the first 6 months of life and AD outcome reported at the age of 18 months. There is some evidence that pHF may be effective in the prevention of allergic disease even in infants without a family history of atopy (51, 52). In contrast to A.R.T. and GINI, the large Melbourne Atopy Cohort Study (MACS) (53), found no evidence to support the use of a pHF on allergy prevention in high-risk infants.

Amidst the longstanding debate (7, 54–56), a number of scientific organizations have recently withdrawn or downgraded their recommendation for pHF in allergy prevention guidelines (57–62). The switch in recommendation was mainly based on the findings of recent systematic reviews and meta-analyses which pointed out inadequate scientific evidence supporting the preventive role of pHF and raised concerns for bias on several levels in most previous studies (including selection, assessment, attrition and conflict of interest) (13, 14). However, since it appears that effectiveness in risk reduction may be dependent of specific physicochemical properties of pHF used (as recognized by EFSA), the outcomes of meta-analyses should be handled with care since these analyses may do not make such a differentiation in pHFs. In 2016, EFSA noted that the safety, suitability and efficacy of each specific formula containing protein hydrolysates has to be established by clinical trials (21). Following this recommendation, and taking into consideration limitations of previous studies, A.R.T. aimed to assess the efficacy of a specific whey-based partially hydrolyzed formula by applying rigorous methodology. This study demonstrated a clear risk reducing effect of the pHF on AD development, whereas *post-hoc* analysis indicates a trend towards a preventive effect against CMPA.

### Strengths and Limitations of the A.R.T. Study

The most important strength of the present study is the double-blinded randomized controlled design (63). Furthermore, only high-risk infants with a documented by a physician family history of allergy could participate. Since subjects were recruited from public and private maternity hospitals/clinics in 3 different European countries providing a representative high-risk population sample, results could be generalizable. Infants had not consumed any formula with intact or partially hydrolyzed proteins prior to allocation and solid foods were allowed after the age of 4 months.

A.R.T. is one of the few studies reporting outcomes at the age of 6 months when CMPA and AD are the most common allergic manifestations (8, 9, 64). All researchers were appropriately trained prior to initiation of the study, and centers were compared during training, to get the most accurate and comparable results. Furthermore, statistical analysis was performed by an independent third party. All the data were prospectively collected and uploaded on the online Research Manager database and locked before analysis. Both the ITT and PP analyses are presented and even though the ITT results are considered more reliable (12), PP results are almost identical further supporting the ART study findings.

Importantly, CMPA was objectively confirmed by open oral food challenge after an elimination diet in accordance with current CMPA guidelines (6, 27, 45, 65–69). Although this could be considered as a strength of the study, one could see it as a limitation since the gold standard for food allergy diagnosis (double-blinded placebo-controlled food challenge) was not performed. It is generally accepted that the clinical diagnosis of AD in early life is challenging as the proposed criteria are difficult to apply (70, 71) given that they have been created for the assessment of older children. However, the AD outcome in ART was defined as a clinical diagnosis of AD by experienced clinicians supported by the objective SCORAD (23) tool and the awareness CoMiSS (24) tool.

There could be arguments that in order to assess the risk reduction effect of pHF the numbers of exclusively formula-fed infants should have been larger and that the protective effect observed in the A.R.T. study may be dependent only on the different maternal bioactive factors in breast milk (72). However, given that the number of mixed-fed infants and the amount of infant formula consumed (Supplementary Table S4) were similar between the two study arms (pHF and SF), the observed risk-reduction effect is likely due to the specific pHF used in the study. Moreover, since it is unethical to randomize breastfeeding and given that exclusive breastfeeding is strongly recommended by WHO (73), a study to enroll high numbers of exclusively formula-fed infants would have been difficult to gain approval by any bioethics committee.

Another limitation is the sample size and power estimation of the study that was based on limited data from older studies. The assumed incidence of CMPA was 20% (30) in the SF group, whereas in the actual study we found only 8%, which might suggest why the study did not detect full effectiveness of the pHF.

The number of subjects who strictly adhered to the study protocol (PP analysis) may sound relatively low [105/160 subjects in the pHF and 120/171 subjects in the SF group (drop-out rate of 34 and 30% respectively)]. However, it is close to what we have expected (estimated drop-out rate 30%) for this type of longitudinal intervention randomized controlled trial applying a demanding protocol. Interestingly, a review of 71 randomized controlled trials published in four prestigious medical journals revealed that 1 in 5 trials had drop-out rates of more than 20% (74).

The ITT analysis is considered more reliable acting as a critical safeguard against bias because it preserves the benefits of randomization which include balancing known and unknown factors and eliminating selection bias (12, 74). According to our estimations, 158 infants had to be included per treatment arm for the ITT analysis and in the A.R.T. study these numbers were achieved (160 in pHF and 171 in SF group) supporting valid results. We could assume that if we had the opportunity to recruit more subjects in each group, we would reasonably expect the observed risk-reduction effects to be even stronger in both the ITT and PP analyses.

Data on the possible mechanisms of action (i.e., immune profiling, Th1/Th2 cytokines, specific IgE measurements) by which a pHF may protect against the development of allergy would have been interesting. However, this was not within the scope of the A.R.T. study as our primary objective was to investigate the potential risk reduction effect with this specific pHF and not to explore the possible underline mechanisms of action which are amply discussed in other publications (75–77) and would be extremely important to incorporate in future studies within this research field.

Finally, true blinding for pHF is difficult due to its specific taste and smell as compared to SF (7, 12). Also working with FCA and FCB codes at the bottom of the tins was not ideal as it may lead to bias. However, products were prepared at home and FCA and FCB were nowhere explained during the study. Only a product developer at FrieslandCampina, not involved in the study, had access to decoding. Code-break or de-blinding did not take place until statistical analyses were completed.

## Conclusion

The data from A.R.T. support that infants at high-risk for allergy who are not exclusively breastfed, may benefit from a nutritional intervention applying mixed-feeding with a specific whey-based pHF compared to mixed-feeding with a standard formula. This combination reduced the development of allergic manifestations within the first 6 months of life, in particular Atopic Dermatitis. The protective effect was strongest for infants with a family history of AD. In addition, mixed-feeding with this pHF may also reduce the risk of cow's milk protein allergy development however, further larger studies are needed to confirm this effect. Long-term follow-up of these infants with objective assessment of allergy outcomes will inform about the potential protective effect of this specific whey-based pHF on allergy prevention in general, and justify the role of this early nutritional intervention.

## Data Availability Statement

The original contributions presented in the study are included in the article/Supplementary Material, further inquiries can be directed to the corresponding author/s.

## Ethics Statement

The studies involving human participants were reviewed and approved by the appropriate independent Ethics Committee in each center as detailed in the Supplementary Material. The study was conducted in accordance with the guidelines of the Declaration of Helsinki and the International Conference on Harmonization guidelines on Good Clinical Practice and registered in the Netherlands Trial Registry [Identifier: Trial NL6120 (NTR6259)]. Written informed consent to participate in this study was provided by the participants' parents/legal guardians.

## Author Contributions

NN, RP, EK, and MS drafted the manuscript. YM, NN, RP, IT-V, LU, UK, and AS have been involved in the design of the study. NN, RP, EK, MS, MM-A, SP, MT, AK, NI, TB, ZI, VP, OS, PX, and EP in the acquisition of data. All authors contributed to the interpretation of the data, reviewed, and agreed to the final version of this article.

## Funding

This research was funded by FrieslandCampina Nederland B.V., which provided the infant formulas and financial support to the participating centers to conduct the study.

## Conflict of Interest

NN, PX, and YM have received an honorarium for the speaker's bureau from FrieslandCampina. IT-V, LU, UK, and AS are employees at FrieslandCampina. The remaining authors declare that the research was conducted in the absence of any commercial or financial relationships that could be construed as a potential conflict of interest.

## Publisher's Note

All claims expressed in this article are solely those of the authors and do not necessarily represent those of their affiliated organizations, or those of the publisher, the editors and the reviewers. Any product that may be evaluated in this article, or claim that may be made by its manufacturer, is not guaranteed or endorsed by the publisher.

## Abbreviations

AAF: Amino-acid based Formula
AD: Atopic Dermatitis
AM: Allergic Manifestations
A.R.T.: Allergy Reduction Trial
BF: Breastfeeding
CI: Confidence Interval
CMP: Cow's Milk Protein
CMPA: Cow's Milk Protein Allergy
CoMiSS: Cow's Milk-related Symptom Score
DBPCFC: Double Blinded Placebo Control Food Challenge
DSMB: Data Safety Monitoring Board
EAACI: European Academy of Allergy and Clinical Immunology
EFSA: European Food Safety Authority
eHF: Extensively Hydrolyzed Formula
GINI: German Infant Nutritional Intervention
ITT: Intention-to-Treat
MACS: Melbourne Atopy Cohort Study
MRAM: Milk Related Allergic Manifestations
NNT: Number Needed to Treat
OFC: Oral Food Challenge
SM: Supplementary Material
pHF: Partially Hydrolyzed Formula
PP: Per-Protocol
RR: Relative Risk
SCORAD: Scoring for Atopic Dermatitis
SF: Standard Formula
SPT: Skin Prick Test.
