# Supplementary material for: The Risk Reduction Effect of a Nutritional Intervention With a Partially Hydrolyzed Whey-Based Formula on Cow's Milk Protein Allergy and Atopic Dermatitis in High-Risk Infants Within the First 6 Months of Life: The Allergy Reduction Trial (A.R.T.), a Multicenter Double-Blinded Randomized Controlled Study
Source: Front Nutr. 2022 May 25;9:863599. doi: 10.3389/fnut.2022.863599 (PMC9174747; doi:10.3389/fnut.2022.863599)
Supplement: Supplementary file 1 [file Data_Sheet_1.pdf]

ONLINE REPOSITORY MATERIAL

METHODS

Study design and participants

The Allergy Reduction Trial (A.R.T.) is a multicenter, double-blinded, parallel, randomized controlled study assessing differences in the incidence of Cow's Milk Protein Allergy (CMPA) [presented as part of milk related allergic manifestations (MRAM) in the original study protocol] and atopic dermatitis (AD) within the first six months of life in apparently healthy term infants at high risk of developing allergy (family history of allergy). Two intervention formulas were provided to study infants, fed exclusively or as supplementary to breastfeeding: a) a partially hydrolyzed whey-based formula (Frisolac Gold preventive HA) or b) an intact protein cow's milk formula (Frisolac Gold; standard formula; SF). Exclusively breastfed infants were also followed as a parallel observational group. The study was conducted in 6 centers in 3 countries; Bulgaria (1), Cyprus (1) and Greece (4) between 2017-2019.

Due to increased dropout and reduced recruitment rate during the first months of the study, the initial protocol was amended to include a lower number of follow-up visits, additional recruiting centers, recruitment-period time extension and sample size re-estimation.

The study protocol, information letter to the parents/legal guardians, and written informed consent form were reviewed and approved by the appropriate independent ethics committee in each center: Bulgaria: Research Ethics Committee of Medical University of Varna; Cyprus: Cyprus National Bioethics Committee; Greece: a) Research Ethics Committee of Alexandra Hospital, b) Research Ethics Committee of Aretaieio Hospital, c) Research Ethics Committee of Attikon Hospital, d) Research Ethics Committee of Helena Hospital. The study was conducted in accordance with the guidelines of the Declaration of

Helsinki and the International Conference on Harmonization (ICH) guidelines on Good Clinical Practice (GCP) and was registered in the Netherlands Trial Registry [Identifier: Trial NL6120 (NTR6259)].

Recruitment procedures

During the 7th – 9th month of gestation (or shortly after delivery), families that were attending public and private maternity clinics, were interviewed by study researchers regarding family history of allergy, using an enriched validated questionnaire[1] in order to identify infants at high-risk of developing allergy. Family history of allergy included Atopic Dermatitis/Eczema, Allergic Asthma, Allergic Rhinitis-Conjunctivitis, Urticarial rash with exposure to pets and Food Allergy in parents and/or siblings. Only cases with a doctor diagnosis of allergic disease in the family were considered to be eligible. Parents were then informed about the A.R.T. study and depending on their willingness to participate, a pre-consent form was requested to be signed. On the day of delivery parents were given the Parent Information Brochure which included detailed information regarding the study procedures. Within four days after delivery, if the inclusion criteria were fulfilled and parents had decided to participate in the study, a consent form was completed.

Inclusion Criteria

Only term infants (≥37weeks), with birth weight ≥2500g, postnatal age <5 days, apparently healthy with no signs of allergy having at least one parent or sibling with confirmed by a doctor history of allergy, who were exclusively breastfed or fed with an extensively hydrolyzed infant formula (eHF) since birth could be recruited. The parents should be willing to bring their infants to the study centers for at least bi-monthly follow-up visits during the first six months (and to answer a telephone questionnaire at the age of 12 months).

Randomization and Treatment Allocation

All mothers were strongly encouraged to breastfeed. However, parents could choose freely the type of their infant's feeding (exclusive breastfeeding, mixed-feeding or exclusive formula-feeding). In case of non-exclusive breastfeeding, subjects were at random allocated to the pHF or the SF as soon as consent was obtained by the parents. An independent statistician created computer-generated randomization tables for each study center. Stratification for gender, type of feeding (exclusively formula-fed or mixed-fed) and presence of AD in a parent and/or sibling was applied.

In case a subject was initially exclusively breastfed and breastfeeding was insufficient or the parents wished to add a complementary infant formula before the age of 10 weeks, then the infant could be allocated to one of the two formula groups.

Study Products, Blinding and Adverse Events

The intervention formulas consisted of a partially hydrolyzed whey-based formula (pHF; Frisolac Gold preventive HA) and an intact protein/standard formula (SF; Frisolac Gold) nutritionally suitable for the first six months of life. Table S1 (Supplementary Material) presents the macronutrients distribution of the two study products. Both are commercially available and are produced in the Netherlands by FrieslandCampina. The formulas were packed in identical white unlabeled 400g tins carrying the description "not for commercial use" and were distinguished by a different code-name (FCA or FCB) printed at the bottom of the tins in a small font-size. This was performed by personnel of the packaging department at FrieslandCampina that were not involved in the study. All study personnel and parents were blinded to the study formulas code-matching groups until the whole study was completed and database was locked. Study products were provided for free to the study participants during the first six months of life.

All adverse events (including those classified as serious) and actions taken to resolve them were recorded throughout the study. An independent Data Safety Monitoring Board (DSMB; 2 persons including a Dutch pediatrician and a Dutch allergist) evaluated and discussed the accumulated adverse events at least twice a year to monitor participant safety and make recommendations concerning the continuation, modification or termination of the trial. Two sealed code-break envelopes (each one corresponding to the respective study formula codes FCA and FCB) were kept securely locked and upon request, the specific envelope would have been made available for de-blinding. Still, no "code-break" requests occurred throughout the study and de-blinding did not take place.

Follow-up evaluation and compliance

Ten days after delivery, the research team contacted the parents by phone to assess the protocol compliance (no formula allowed except the study formulas). Infants visited the study centers for follow-up assessments bi-monthly (at 2nd, 4th and 6th month) during the first six months of life. Optional follow-ups were performed at the age of one and three months and additional visits were performed at any time point if needed (development of any signs of allergy or adverse events). Infants had not consumed any other formula prior to allocation and solid foods were allowed only after the age of 4 months. No dietary restrictions were advised to breastfeeding mothers.

Formula intake compliance was evaluated using a 7-day milk diary completed during the week preceding the 1st, 2nd, 4th and 6th month of age. At 1 month of age, milk intake was reported via phone calls (or during an optional follow up visit). If formula consumption was less than 40 mL/kg body weight/day during the 1st month of life or less than 60 mL/kg body weight/day during the 2nd month (and thereafter), then the infant was considered as a dropout. These cut-off values assured a formula intake of about 25% and 40%,

respectively, of total daily milk intake[2]. For infants that were initially exclusively breastfed and allocated to a study formula before the age of 10 weeks, the same formula quantity requirements were applied.

At the follow-up visits, infants were clinically examined by experienced pediatricians and nurses for the presence of CMPA and AD. Suggestive CMPA and AD symptoms were objectively scored across centers using the Scoring for Atopic Dermatitis (SCORAD[3]) tool and the awareness Cow's Milk-related Symptom Score (CoMiSS[4,5]) tool. SCORAD comprises skin symptoms (location, extend and severity of erythema, oedema, oozing, excoriations lichenifications, dryness and subjective symptoms), and CoMiSS comprises crying, regurgitations, stools, skin and respiratory symptoms. Furthermore, the "Screening for IgE- and non-IgE-mediated food allergy symptoms questionnaire" was completed[6]. CMPA was confirmed by oral food challenge by a pediatrician or a pediatric allergist. When an infant was diagnosed with CMPA, management according to the individual national health care system in each country was applied, ensuring that the affected subject would receive the appropriate hypoallergenic formula.

Anthropometric measurements focused on weight, length and head circumference were performed by the same two well-trained research team members at each center, using calibrated digital infant scales (SECA 354) with a precision of +/- 20g for weights below 20Kg, an infantometer (SECA 210) measuring to the nearest 0.1cm and a non-elastic tape (SECA 211) measuring to the nearest 0.1cm, respectively. All measurements were performed in triplicates and averaged. In case a pair-wise difference between the three measures was >100g for weight, >0.7cm for length and >0.5cm for head circumference then, a 4th measurement was performed and the three nearest measurements were written averaged.

Reducing intra- and inter-observer variation

Before the launch of the study, the research team was trained by an allergy specialist on how to apply both SCORAD and CoMiSS tools to evaluate and score possible atopic dermatitis and allergy symptoms in

order to reduce intra- and inter-observer variation. In addition, pediatricians and nurses practiced on performing accurate anthropometric measurements.

Definition of study Outcomes

Two primary outcomes were defined: CMPA (as part of MRAM) and AD. The definitions of CMPA and AD are described in detail in the main manuscript. As CMPA and AD are the most common allergic manifestations presenting in the first 6 months of life no other MRAM were considered in the statistical analysis at this time-point.

Milk related allergic manifestations (MRAM)

MRAM was defined as presence of AD and/or allergic urticarial rash and/or gastro-intestinal manifestations (assessed objectively as total CoMiSS score ≥ 12 or CoMiSS score for Skin Symptoms on Urticaria ≥ 1 or CoMiSS score for Respiratory symptoms ≥ 1) combined with a confirmation of CMPA based on positive oral food challenge (OFC) or suspected CMPA based on clinical evaluation but with incomplete OFC (due to parents' refusal to proceed with the challenge).

Reasons for dropping-out (early termination)

Infants were considered as dropouts if a) parents withdrew consent (and they were allowed to do so at any time-point of the study if they wished), b) infant was fed with any other than the allocated study formula (or an eHF/AAF), c) an exclusively breastfed infant switched to mixed-feeding after the age of 10 weeks, d) formula intake during the 1st and 2nd months of life was less than 40 and 60mL/KgBW/d respectively, e) weaning foods were introduced before the age of 4 months, and f) infant had experienced a Severe Adverse Event.

Sample size estimation

The sample size calculation was based on two studies: the GINI study of Von Berg et al. (2003)[1], and a study performed by Halken et al. (2000)[7], called ‘the Halken study’. Furthermore, the practical limitation that all high-risk infants had to be recruited within 1 to 1.5 years was taken into account. Working with the data from GINI and Halken, the anticipated incidence in the SF group was estimated at 16% vs. 5% (4.7% rounded) in the pHF group, and thus a protective effect size of 68% was anticipated. Using a significance error of 5% (2-tailed) and power of 80%, a sample size of 121 infants per treatment arm should be available for evaluation. Assuming a drop-out rate of 30%, 158 infants had to be included per treatment arm.

REFERENCES

1. Von Berg A, Koletzko S, Grübl A, Filipiak-Pittroff B, Wichmann HE, Bauer CP, et al. The effect of hydrolyzed cow's milk formula for allergy prevention in the first year of life: The German Infant Nutritional Intervention Study, a randomized double-blind trial. J Allergy Clin Immunol. 2003;111:533–40.

2. Dixon M, Crawford D, Teasdale D, Murphy J. Nursing the highly dependent child or infant. A John Wiley & Sons, Ltd, Publication; 2009. ISBN: 1405151765

3. Stalder JF, Taïeb A, Atherton DJ, Bieber P, Bonifazi E, Broberg A, et al. Severity scoring of atopic dermatitis: The SCORAD index: Consensus report of the european task force on atopic dermatitis. Dermatology. 1993;186:23–31.

4. Nestlé Health Science. Cow's Milk-related Symptom Score CoMiSS An awareness tool. 2015; Available from: https://www.nestlehealthscience.co.uk/sites/default/files/hcp-education-hub/cma/documents/comiss brochure web.pdf

5. Vandenplas Y, Dupont C, Eigenmann P, Host A, Kuitunen M, Ribes-Koninckx C, et al. A workshop report on the development of the Cow's Milk-related Symptom Score awareness tool for young children. Acta Paediatr Int J Paediatr. 2015;104:334–9.

6. Grabenhenrich LB, Reich A, Bellach J, Trendelenburg V, Sprikkelman AB, Roberts G, et al. A new framework for the documentation and interpretation of oral food challenges in population-based and clinical research. Allergy Eur J Allergy Clin Immunol. 2017;72:453–61.

7. Halken S, Hansen KS, Jacobsen HP, Estmann A, Christensen AEF, Hansen LG, et al. Comparison of a partially hydrolyzed infant formula with two extensively hydrolyzed for allergy prevention: A prospective, randomized study. Pediatr Allergy Immunol. 2000;11:149–61.

Table S1 – Simplified composition of the study formulas (per 100mL)

	**pHF**	**SF**
Protein (g)		1.4
Protein hydrolysate (g)	1.6	
Fat (g)<br>DHA (mg)<br>AA (mg)	3.5<br>6.9<br>6.9	3.5<br>6.9<br>6.9
Carbohydrates (g)<br>Lactose (g)<br>Glucose, maltose, starch, sucrose (g)	7.0<br>6.5<br>0.5	7.0<br>6.5<br>0.5
Dietary fiber:<br>Galactooligosaccharides (g)	0.17	2.7
Energy (kcal/100ml)	66	66
Na (mg)	20	23
K (mg)	65	79
Ca (mg)	50	56
Mg (mg)	6	6.4
P (mg)	30	31
Fe (mg)	0.78	0.77
Vitamin A (µg-RE)	70	74
Vitamin D (µg)	1.2	1.1

RESULTS

Table S2 - The Incidence and Relative Risk for CMPA and AD and within the first six months of life in mixed-fed infants including post-hoc analysis

			ITT analysis set				***PP analysis set***			
			pHF (N=143)	***SF (N=152)***	***RR (95% CI)***	***p-value***	***pHF (N=89)***	***SF (N=106)***	***RR (95% CI)***	***p-value***
**Model 1**		**CMPA, n (%)**	5 (3.5)	12 (7.9)	0.44 (0.17, 1.20)	0.11	4 (4.5)	10 (9.4)	0.56 (0.19, 1.63)	0.29
		**CMPA+SPT, n (%)**	6 (4.2)	14 (9.2)	0.45 (0.18, 1.12)	0.084	6 (6.7)	14 (13.2)	0.56 (0.23, 1.38)	0.21
		**CMPA+susp, n (%)**	7 (4.9)	16 (10.5)	0.46 (0.20, 1.06)	0.067	6 (6.7)	14 (13.2)	0.56 (0.23, 1.38)	0.21
		**AD, n (%)**	15 (10.5)	30 (19.7)	0.50 (0.29, 0.88)	**0.016**	10 (11.2)	27 (25.5)	0.47 (0.24, 0.91)	**0.024**
**Model 2**	**FHAD+**	**n**	**41**	**41**			**22**	**31**		
		**CMPA, n (%)**	2 (4.9)	6 (14.6)	0.37 (0.08, 1.68)	0.20	2 (9.1)	4 (12.9)	0.82 (0.18, 3.66)	0.79
		**CMPA+SPT, n (%)**	3 (7.3)	5 (12.2)	0.63 (0.16, 2.49)	0.51	3 (13.6)	5 (16.1)	0.91 (0.24, 3.40)	0.89
		**CMPA+susp, n (%)**	3 (7.3)	7 (17.1)	0.46 (0.13, 1.62)	0.23	3 (13.6)	5 (16.1)	0.91 (0.24, 3.40)	0.89
		**AD, n (%)**	3 (7.3)	12 (29.3)	0.24 (0.08, 0.79)	**0.019**	2 (9.1)	10 (32.3)	0.31 (0.08, 1.25)	0.10
	**FHAD-**									
		**n**	**102**	**111**			**67**	**75**		
		**CMPA, n (%)**	3 (2.9)	6 (5.4)	0.51 (0.13, 1.93)	0.32	2 (3.0)	6 (8.0)	0.42 (0.09, 1.95)	0.27
		**CMPA+SPT, n (%)**	3 (2.9)	9 (8.1)	0.34 (0.10, 1.23)	0.10	3 (4.5)	9 (12.0)	0.40 (0.12, 1.37)	0.14
		**CMPA+susp, n (%)**	4 (3.9)	9 (8.1)	0.45 (0.15, 1.40)	0.17	3 (4.5)	9 (12.0)	0.40 (0.12, 1.37)	0.14
		**AD, n (%)**	12 (11.8)	18 (16.2)	0.68 (0.35, 1.32)	0.25	8 (11.9)	17 (22.7)	0.54 (0.26, 1.15)	0.11

Poisson generalized estimating equation (GEE) regression analysis. Model 1 adjusts for study formula and stratification factors: country, gender and FHAD. Model 2 additionally adjusts for the interaction between FHAD and study formula. Figures in bold indicate statistically significant p-values.

***AD:** atopic dermatitis; **CMPA:** cow's milk protein allergy confirmed by oral food challenge (OFC); **CMPA+SPT:** CMPA plus IgE-mediated CMPA cases considered as CMPA based on clinical history and positive Skin Prick Test to cow's milk without performing OFC; **CMPA+susp:** CMPA and CMPA+SPT plus clinically suspected cases of CMPA in which symptoms resolved after introducing an extensively hydrolyzed formula, but parents did not consent for OFC; **pHF:** partially hydrolysed formula; **SF:** standard formula; **ITT:** intention-to-treat; **PP:** per-protocol; **RR:** relative risk; **FHAD+:** Family history of AD; **FHAD-:** No family history of AD*

Table S3 - The Incidence for CMPA and AD within six months of life in exclusively formula-fed infants

	ITT analysis set			***PP analysis set***		
	pHF (N=17)	***SF (N=19)***	****p-value***	***pHF (N=16)***	***SF (N=14)***	****p-value***
**CMPA, n (%)**	1 (5.9)	0 (0.00)	0.472	1 (6.3)	0 (0.00)	> 0.99
**AD, n (%)**	2 (11.8)	2 (10.5)	> 0.99	2 (12.5)	2 (14.3)	> 0.99

**Fisher's exact test*

***AD:** atopic dermatitis; **CMPA:** cow's milk protein allergy confirmed by oral food challenge (OFC); **pHF:** partially hydrolysed formula; **SF:** standard formula; **ITT:** intention-to-treat; **PP:** per-protocol; **RR:** relative risk*

Table S4: Mean daily formula consumption reported at each bi-monthly follow-up visit in both exclusively formula-fed and mixed-fed infants

	ITT analysis set			***PP analysis set***		
Daily formula intake, mL, mean (SD)	***pHF (N=160)***	***SF (N=171)***	****p-value***	***pHF (N=105)***	***SF (N=120)***	****p-value***
**2nd month follow-up**	629.1 (261.5)	653.9 (251.5)	0.43	696.8 (233.1)	704.9 (217.4)	0.79
**4th month follow-up**	780.7 (238.7)	819.0 (222.8)	0.20	811.4 (201.9)	839.1 (207.2)	0.33
**6th month follow-up**	730.6 (256.9)	787.7 (240.6)	0.09	737.2 (256.9)	803.5 (239.5)	0.06

**p-value derived from the Independent Samples T-Test*

***pHF:** partially hydrolysed formula; **SF:** standard formula; **ITT:** intention-to-treat; **PP:** per-protocol;*
***N**: number of subjects in analysis population; **SD**: Standard Deviation*
